# Replication Strategy for Spatiotemporal Data Based on Distributed Caching System

**DOI:** 10.3390/s18010222

**Published:** 2018-01-14

**Authors:** Lian Xiong, Liu Yang, Yang Tao, Juan Xu, Lun Zhao

**Affiliations:** School of Communication and Information Engineering, Chongqing University of Posts and Telecommunications, Chongqing 400065, China; yangliu@cqupt.edu.cn (L.Y.); S160131179@stu.cqupt.edu.cn (J.X.); zhaolun@cqupt.edu.cn (L.Z.)

**Keywords:** replica, spatiotemporal date, spatiotemporal locality and correlation, distributed cache, smart city

## Abstract

The replica strategy in distributed cache can effectively reduce user access delay and improve system performance. However, developing a replica strategy suitable for varied application scenarios is still quite challenging, owing to differences in user access behavior and preferences. In this paper, a replication strategy for spatiotemporal data (RSSD) based on a distributed caching system is proposed. By taking advantage of the spatiotemporal locality and correlation of user access, RSSD mines high popularity and associated files from historical user access information, and then generates replicas and selects appropriate cache node for placement. Experimental results show that the RSSD algorithm is simple and efficient, and succeeds in significantly reducing user access delay.

## 1. Introduction

Among recent advancements in technology, cloud computing and the Internet of Things are widely applied in a smart city. As a result, massive spatiotemporal data with location, time, and type attributes will be produced, such as meteorological data, hydrological data, natural disaster data, and remote-sensing images. Such data are usually characterized by wide variety, large amount, high redundancy, and dynamic growth over time [1,2]. A smart city can quickly and conveniently provide users with rich predefined applications through a network platform based on the users’ demands for spatiotemporal data services, such as data visualization, spatiotemporal associated analysis, temporal emergency aid, and massive information retrieval.

Under data intensive and access intensive scenarios in a smart city, the traditional single node cache cannot meet the requirements in storage capacity and processing speed. Studies have shown that the distributed cache method can minimize network delay and enhance data access speed [3,4]. The replica strategy can further improve the performance of distributed cache and reduce access delay for systems with the same infrastructure, node performance, network bandwidth, and associated features.

The core of any replica strategy is cache file selection and replica creation placement. Selecting the appropriate file to be placed in the cache can effectively improve the cache hit rate. The queue probability of access requests can be reduced effectively by generating replicas and placing them in the appropriate cache nodes such that the request process can be accelerated. The higher the cache hit rate, the smaller the request processing time, and the better the cache performance.

However, the spatiotemporal data of user access in a smart city usually has spatiotemporal locality and correlation [5]. For example, if a user checks the weather conditions in a certain area, the change trend of this area for next week may be surveyed as well. When searching for nearby hotel information, related traffic information may also be queried. Specifically, if we place those files that are frequently accessed by users into different cache nodes, user access delay will depend not only on the queuing time, but also on the cross-node scheduling time. Most existing replica strategies focus on improving the cache hit ratio and load balancing that can effectively reduce the queuing time. However, cross-node scheduling time is rarely addressed.

In this paper, we propose a replication strategy for spatiotemporal data (RSSD) based on distributed caching system in a smart city. RSSD takes advantage of the spatiotemporal locality and correlation of user access to mine high popularity and associated files from historical users’ access information to generate replica for these files and select appropriate cache node for placement on the premise of load balance, aimed at achieving bidirectional optimization of access request of queuing and cross node scheduling time.

The rest of this paper is organized as follows: Section 2 presents the current research status of performance optimization of distributed caching at home and abroad. Section 3 introduces the system model while Section 4 describes the implementation of our replica strategy. Section 5 presents and discusses the performance evaluation results of our replica strategy. Finally, Section 6 briefly summarizes our findings and concludes the paper.

## 2. Related Works

To date, several studies on replica mechanisms in cluster environments have been carried out. In this section, we will provide a review of replica mechanisms which are most related to our work.

Tang et al. [6,7] identified hot files in a data grid by counting the number of file accesses in historical access information and created replica to cache these files. In order to improve the resource access efficiency in P2P networks, Sun et al. [8] cache the files with high access frequency and long average response time by using a predefined global average expectation time to determine the number of replica. Then, the optimal placement of the copy is calculated based on the access frequency, node load, and real-time bandwidth. In order to improve the WebGIS response speed, Li et al. [9] selected a cache file and calculated the number of replica according to the total capacity of the cache server and file access probability; then, the usage of each cache node was deduced to determine the location of the replica files. Chang et al. [10] proposed that files with high popularity will be more probably accessed in the future; therefore, the number of accesses to the file was weighted based on the access time. The closer the current time point is, the greater the weight will be. Then, the number of replica was determined by the number of files accessed, and the copy was placed in the most frequently accessed nodes. Sun et al. [11] also used a time weighting method for access times and for selecting access to hot files for caching. A similar approach was also presented in [12]. Pan et al. [13] proposed a dynamic replication management strategy in distributed GIS, where an enhanced Q-value scheme to calculate the number of copies for each replica and a copies placement strategy based on probability of replica are designed.

Wei et al. [14] calculated the number of replica according to the minimum availability of files in the cloud storage system in terms of availability of the system. Then, the placement of replica was determined by using the capacity of data nodes and the availability of data blocks. Li et al. [15] constructed an access cost graph by combining information of user requests and network distance. A modified Dijkstra’s algorithm was introduced to search for the shortest path in the access cost graph, which corresponds to an optimal cache deployment for the system. Tu et al. [16] treated the network topology of distributed system as a tree, and each node in the tree corresponds to a data server. Then, the shortest path node was calculated to meet the need of data request, and the access was placed such that it corresponds to the adjacent nodes of the tree. According to the request frequency and system capacity of data objects, Zaman et al. [17] proposed a distributed greedy replica placement mechanism aimed at reducing the average access time of data objects. Nagarajan et al. [18] proposed a prediction-based replication strategy for data-intensive applications—Intelligent Replica Manager (IRM)—designed and incorporated in the middleware of the grid for scheduling data-intensive applications.

Lin et al. [19] considered that different applications have different QoS requirements in cloud computing systems and proposed a greedy algorithm called QoS-aware data replication (QADR) to minimize the replication cost and the number of replica. The principle of this QADR is that applications with higher QoS requirements should be given priority for file replication. Similarly, a replication that considers system application QoS requirements was also studied in [20,21,22]. In addition, Tos et al. [23] classified and summarized existing dynamic replica algorithms based on data grids, and highlighted the advantages and disadvantages of each strategy and the applicable scenarios. Suciu et al. [24] proposed a collaborative monitoring software platform named MobiWay for big data in a smart city, aiming at supporting Intelligent Transportation Systems (ITS) applications by sharing open traffic data and acting as a middleware connection hub.

From the above studies, we can observe that the study of current replica mechanism mainly focused on the file size, access frequency and probability, average response time, system service capability, capacity of node, the real-time bandwidth and other information aimed at generated replica and selecting the appropriate node for placement. These methods can also be used in distributed cache system to improve cache hit rate and balance node burden.

However, since the spatiotemporal data of user access in a smart city often has obvious spatiotemporal locality and correlation, the user access latency is not only related to the cache hit ratio and node load, it is also closely related to cache file placement. Therefore, this paper aims to develop a reasonable replica strategy for the purpose of achieving bidirectional optimization of access request queuing and cross node scheduling time while enhancing cache hit rate, improving the performance of distributed caching, and reducing user access delay.

## 3. System Model

**Figure 1:**
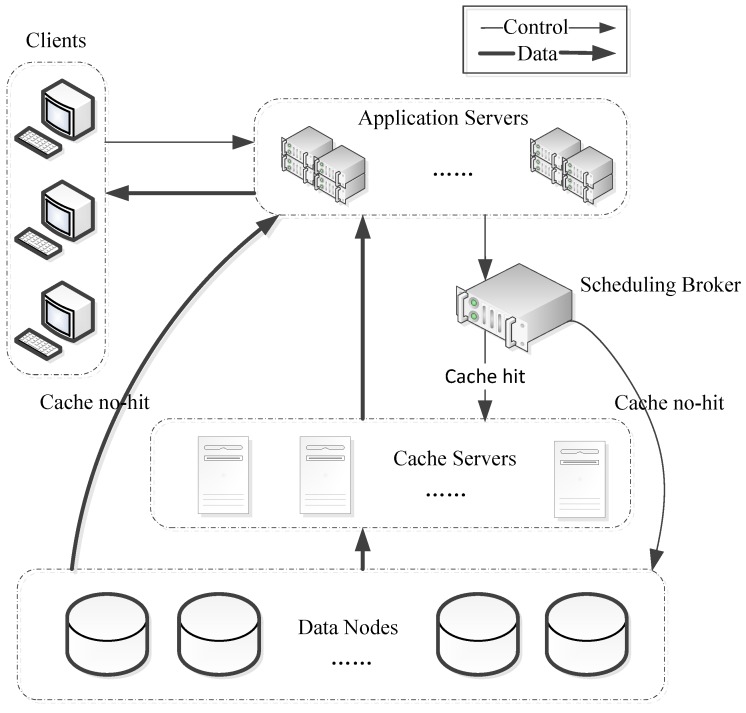
Application service system architecture of smart city.

Figure 1 shows a typical distributed caching-based application service system architecture of a smart city, which consists of five parts: clients, application servers, scheduling broker, cache servers, and data storage nodes. 

All spatiotemporal data are stored in data nodes in a distributed manner. The application servers, scheduling broker, cache servers, and data storage nodes are connected via LAN and clients are connected through the Internet. When a client needs a file, it will first check for the file in the nearest cache node. If there is no such file in the cache node, then the client will choose to read from the data nodes.

So it is necessary to improve the cache hit rate, as well as reduce the request queuing time and cross node scheduling time for the purpose of accelerating the user access request. To address these three aspects, our solutions are described below.

- Cache files selection: select the files with high popularity according to the access frequency and then place them into the cache. Thus, the cache hit rate can be improved, which insures the files to be obtained from the high-performance cache server as much as possible.
- Replica generation: generate replica according to the popularity of cache file and the capacity of cache nodes, then, distribute them into different cache nodes. Hence, the request queuing time can be reduced by balancing the system burden.
- Replica placement: place the replica of access associated file as a whole in the same cache node according to the spatiotemporal correction of user’s access request and the real-time status of the cache node so that the request cross node scheduling time can be reduced.

## 4. Methodology

The RSSD algorithm mainly consists of three parts: cache files selection, replica creation, and replica placement.

### 4.1. Basic Concepts and Methods

#### 4.1.1. Popularity of Files

Studies [10,11] have shown that file access has temporal locality, and its popularity will gradually decrease over time. The more remote access information from the current point of time is, the less impact on the popularity of the file. Therefore, in order to calculate the popularity of files, we segmented historical user access information based on the access time, and created statistics about the file visits in each segment. Then, according to the distance between the segment and the current time point, the weighted visits of the file were calculated as the result of the popularity of the files. We chose files with high popularity as cache files.

Suppose the historical user access information contains the filesets *F_set_* = {*f*_1_, *f*_2_, …, *f_N_*} during the observation time *T* = [*T_begin_*:*T_end_*]. We divide *T* into *N_t_* = (*t_end_* − *t_begin_*)/Δ*t* segments by adopting a time interval Δ*t* first, and the *t*, 1 ≤ *t* ≤ *N_t_* segment is [*t_begin_* + (*t* − 1) × Δ*t*, *t_begin_* + *t* + Δ*t*]. Assuming that the visits of file *f_n_*, 1 ≤ *n* ≤ *N* in the time of *t* segment is *a_n,t_*, then the popularity of file *f_n_* can be expressed as:

(1) $$\xi_{n}=\sum_{t=1}^{N_{t}}a_{n,t}\times e^{-\left(N_{t}-t\right)}/N_{t}$$

where $e^{-\left(N_{t}-t\right)}$ is time varying weights and *e* is a mathematical constant. The average popularity of the filesets *F_set_* can be expressed as:

(2) $$\bar{\xi }=\sum_{n=1}^{N}\xi_{n}/N$$

It is obvious that if some files are always accessed simultaneously by a user within a limited period of time, and the access frequency is greater than the predefined threshold, then these files are access associated (in Section 4.1.3, we define this threshold as the average access popularity $\bar{\xi }$). Therefore, define the popularity of associated file to be the same as the popularity of a single file. Assuming that the number of files *f_n_*, 1 ≤ *n* ≤ *N* accessed in the time of *t* segment is *a_n,t_*, then the popularity of access of associated files, which consists of *k* files of filesets $F_{set}^{k}$ can be expressed as:

(3) $$\xi^{k}=\sum_{t=1}^{N_{t}}\min(a_{i,t},\;a_{j,t},\;\cdots ,\;a_{k,t})\times e^{-\left(N_{t}-t\right)}/\left(k\times N_{t}\right)$$

where min(*a*_1,*t*_, *a*_2,*t*_, …, *a_k,t_*) is the minimum number of files accessed in the filesets $F_{set}^{k}$ during the time of *t*, 1 ≤ *t* ≤ *N_t_* segment. For example, the number of files accessed is *a*_1,*t*_ = 3, *a*_2,*t*_ = 2, *a*_3*,t*_ = 6 in the filesets {*f*_1_,*f*_2_,*f_3_*} during *t*, 1 ≤ *t* ≤ *N_t_* segment, then the minimum number of files accessed is min(*a*_1,*t*_, *a*_2,*t*_, *a*_3*,t*_) = *a*_2,*t*_ = 2, and the popularity of accessing associated files is $\xi^{k}=\xi^{3}=\sum_{t=1}^{1}2\times e^{-\left(1-t\right)}/\left(3\times 1\right)=$ 2/3.

#### 4.1.2. Q-Value Scheme for Cache Replica Generation

How to create a certain number of replica for each file based on the access popularity in a limited cache server capacity is equivalent to the problem of how to allocate limited seats to different classes according to the number of students in each class. The classical Q-value scheme in the allocation problem can be employed to solve the above problems effectively [9].

Assuming that the distributed cache consists of *L* cache services, the capacity of the *l*, 1 ≤ *l* ≤ *L* cache node *Cache_l_* is *CS_l_*. The cache filesets selected from the filesets *F_set_* based on popularity is *F_cache_* = {*f*_1_, *f*_2_, …, *f_M_*}, where the popularity of cache file *f_m_*, 1 ≤ *m* ≤ *M* is $\xi_{m}$, the number of replica of the file is *R_m_* and the size is *C_m_*. Therefore, if we take the system’s cache capacity as the total number of seats, the popularity of cache file as the number of students in each class, and take the number of replica as the number of seats in each class, then, according to the Q-value method, the Q-value of the cache file *f_m_* can be expressed as:

(4) $$Q_{m}=\xi_{m}{}^{2}/\left(R_{m}\times \left(R_{m}+1\right)\right)$$

Considering that there is no effect of reducing the queuing time of the request if a multiple replica of the same file is placed in the same cache node, then, only one replica of the file is placed in each cache node, that is *R_m_* ≤ *L*. The number of replica of the cache file is calculated as follows:

(1) Create a replica for each cache file in the cache filesets *F_cache_*, that is, *R*_1_ = *R*_2_ =…= *R_M_* = 1; then, the remaining cache space capacity of the system is $CSY_{re}=\sum_{l=1}^{L}CS_{l}-\sum_{m=1}^{M}C_{m}$.
(2) To calculate the Q-value {*Q*_1_, *Q*_2_, …, *Q_M_*} for each cache file, if *Q_m_* = max(*Q*_1_, *Q*_2_, …, *Q_M_*), then add 1 to the replica of file *f_m_*, that is *R_m_* = *R_m_* + 1; then, the remaining cache space capacity of the system is *CSY_re_* = *CSY_re_* − *C_m_*.
(3) Loop step (2) until the remaining cache space of system *CSY_re_* is less than the size of file *f_m_*.
(4) Finally, if the number of replica of the file *f_m_* is more than the number of cached nodes, that is *R_m_* > *L*, then, delete the redundant replica and set the number of replica as *R_m_* = *L*. Delete the file *f_m_* from the filesets *F_cache_*.
(5) Loop steps (2–4), until the remaining cache space *CSY_re_* cannot store any cache files.

#### 4.1.3. Mining Associated Files

Frequent pattern mining algorithm FP-growth can mine frequent subsequences from a given sequence [25,26]. Therefore, if we take the filesets *F_set_* = {*f*_1_, *f*_2_, …, *f_N_*}, which a user has accessed during the observation time *T* = [*t_begin_*:*t_end_*] as a sequence, the file sequence accessed by a user within a time slice Δ*t* is a record in the FP-growth algorithm; if we define the minimum support threshold as the average access popularity of a file *min_sup =* $\bar{\xi }$, then we can use FP-growth to mine frequent files and filesets, and the files in the collection are the access associate files.

• Regional meshing

In addition, considering the efficiency of mining, we further exploit the spatiotemporal locality of user access such that only the files belonging to “the same time period” and “the adjacent geographic region” can be mined. “The same time period” indicates that the access time of the file is during *t*, 1 ≤ *t* ≤ *N_t_* segment, and “the adjacent geographic region” indicates that the location attribute of the file is located at the same geographical range. By narrowing the scope of time and space, the efficiency of mining can be improved.

Suppose that the geographic area is a two-dimensional Euclidean rectangular space [0, X][0, Y], we divide it into *row* × *col* rectangular cells with coding, where the code of the area covered by the *i*-th row *j*-th column is *g_ij_* = *j* + *col* × (*i*−1). Then, for any spatiotemporal data file with three basic attributes, namely, location, time, and type, we assume that it belongs to the cell if it satisfies the following equation:

(5) $$\left\{ \begin{array}{l} (i-1)\frac{X}{row}\leq x\leq i\frac{X}{row},\;1\leq i\leq row\\ (j-1)\frac{Y}{col}\leq y\leq j\frac{Y}{col},1\leq j\leq col \end{array}\right.$$

**Figure 2:**
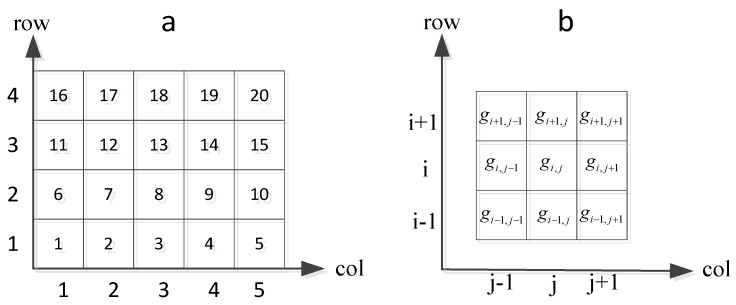
Regional meshing and coding: (**a**) meshing cell coding, (**b**) neighbor cells of *g_ij_*.

Figure 2a shows the geographic rectangular area, which is divided into 4 × 5 cells and the meshing cell coding. Figure 2b shows all the neighbor cells of cell *g_ij_*.

• Mining method

The purpose of this paragraph is to describe how to use the FP-growth algorithm to mine the access associated file $F_{1}^{g_{ij}}$ and non-associated filesets $F_{2}^{g_{ij}}$, which belong to the geographical range of the cell *g_ij_*. The steps are as follows:

*Step 1*: Mine the user access request cache filesets $F_{set}^{g_{ij}}$ that belong to the cell *g_ij_* and its neighbor cells from the user access filesets *F_set_*.
*Step 2*: Calculate the access popularity of each file in $F_{set}^{g_{ij}}$, and find the most popular file *f_k_*, and its popularity $\xi_{k}$.
*Step 3*: The FP-growth algorithm is used to iterate through the user access filesets *F_set_* (the file accessed in a time segment Δ*t* corresponds to a record in the FP-growth algorithm), and the popularity between the file *f_k_* and other files in $F_{set}^{g_{ij}}$ is calculated; if the popularity of access associated between them is greater than or equal to $\bar{\xi }$, then consider these files as access associated.
*Step 4*: The files related to *f_k_* constitute access related filesets; place the files into the filesets $F_{1}^{g_{ij}}$, and then delete these files from $F_{set}^{g_{ij}}$.
*Step 5*: Loop steps (2–5) until there are no access associated filesets generated, and finally the total set $F_{1}^{g_{ij}}$ is formed.
*Step 6*: Files that have low access associated popularity, but with popularity higher than the average access popularity $\bar{\xi }$ are called non-associated files; place these files into the non-associated filesets $\;F_{2}^{g_{ij}}$.

Through the above steps, we can get the access associated filesets $F_{1}^{g_{ij}}$ and non-associated filesets $F_{2}^{g_{ij}}$, and all these files belong to the geographical range of the cell *g_ij_*. Finally, looping through each cell (including *row* × *col* cells) that belongs to the geographical area of the rectangular space [0,*X*][0,*Y*], we can mine all the access associated filesets $F_{1}=\cup_{i=1}^{row}\cup_{j=1}^{col}F_{1}^{g_{ij}}$ and non-associated filesets $F_{2}=\cup_{i=1}^{row}\cup_{j=1}^{col}F_{2}^{g_{ij}}$.

In reality, only the cache file larger than the minimum support threshold *min_sup* = $\bar{\xi }$ will enter the candidate frequent itemsets according to the algorithm principle of FP-growth. Therefore, the essence of mining is to merge and classify the cache filesets *F_cache_* based on the correlation of user access, that is, *F_cache_* = $F_{1}\cup F_{2}$. 

### 4.2. RSSD Algorithm

The basic idea of the RSSD algorithm is mining access associated files from the historical user access information using the user’s access characteristics and file access popularity. Next, place the access associated file as a whole in the same cache node and ensure system load balance according to the storage load, access load, real time bandwidth, and other information of the cache node. 

#### 4.2.1. Cache Files Selection

We cache files with greater popularity than the average popularity; for example, the popularity of files $\xi_{n}\geq \bar{\xi }$. Therefore, all access associated filesets *F*_1_ and non-associated filesets *F*_2_ mined in the Section 4.1.3 will be used as cache files, but the number of replica generated for them needs further calculation.

#### 4.2.2. Replica Generation

Given that the popularity and number of replica per file are not the same, creating replica for filesets is still a great challenge. Therefore, we first used the Q-value scheme to calculate the number of replica for each file, and then split the access associated filesets. Finally, the number of replica of each subset will be calculated. The details are as follows:

(1) Use the Q-value scheme to generate replica for each file in *F_cache_*.
(2) Take any fileset from *F*_1_, and arrange them in descending order according to the number of replica of the file assuming that the sorted filesets are {*f_i_*, *f_j_*, …, *f_k_*}, where *R_i_* ≥ *R_j_* ≥…≥ *R_k_*.
(3) Split the filesets {*f_i_*,*f_j_*, …, *f_k_*} into subsets based on the number of replica of each file. The first subset formed after splitting is itself, that is {*f_i_*, *f_j_*, …, *f_k_*}, and the number of replica is *R*_(*i,j,…,k*)_ = min(*R_i_*, *R_j_*, …, *R_k_*) = *R_k_*. Then, put the subset into the new associated filesets $F_{1}^{\prime }=F_{1}^{\prime }\cup \left\{f_{i},f_{j},\ldots ,f_{k}\right\}$, and record the number of replica *R*_(*i,j,…,k*)_ = *R_k_*.
(4) At this point, the remaining replica of each file in the filesets {*f_i_*, *f_j_*, …, *f_k_*} are *R_i_* = *R_i_* – *R_k_*, *R_j_* = *R_j_* – *R_k_*,…, *R_k_* = *R_k_* – *R_k_*. Then, we delete the files $f_{z}^{g_{ij}}$ with zero copy number to form a new fileset {*f_i_*, *f_j_*, …, *f_k_*} = {*f_i_*, *f_j_*, …, *f_k_*} − *f_k_*.
(5) Repeat steps (2–4) until the filesets {*f_i_*, *f_j_*, …, *f_k_*} are split. To this extent, the number of files in the filesets is zero, or there is only one file *f_i_* with the largest number of replica. For the remaining file *f_i_*, we treat it as non-associated file (at least two files can be referred to as associated) and put them into the non-associated filesets $F_{2}=F_{2}\cup f_{i}$, and record the number of replica *R_i_* = *R_i_* – *R_j_* −…− *R_k_*.
(6) Loop steps (2–5) until every MAF is split in the filesets *F*_1_, and put the results of the split into the filesets $F_{1}^{\prime }$ and *F*_2_. Similarly, here are $F_{cache}=F_{1}^{\prime }\cup F_{2}$.

Here is an example of the process described above. Suppose that the access related filesets {*f_i_*, *f_j_*, …, *f_k_*} are {*f*_1_, *f*_2_, *f*_3_} in descending order according to the number of replica, and the number of replica of each file is *R*_1_ = 5, *R*_2_ = 3, *R*_3_ = 1. Then, according to previous steps, the first subset is $\left\{f_{1}^{g_{ij}},f_{2}^{g_{ij}},f_{3}^{g_{ij}}\right\}$, and the number of replica is *R*_(1,2,3)_ = *R*_3_ = 1. The second subset is {*f*_1_,*f*_2_}, and the number of replica is *R*_(1,2)_ = *R*_2_ − *R*_3_ = 2. For the last remaining single file *f*_1_, we treat it as a non-associated file, and the number of replica is *R*_1_ = *R*_1_ − *R*_2_ − *R_3_* = 1.

After the above steps, we can calculate the new access associated filesets $F_{1}^{\prime }$ and create a copy for each subset, and a copy of the file in the non-associated filesets *F*_2_ can also be created.

#### 4.2.3. Replica Placement

Taking the access associated file as a whole and placing it into the same cache node results in an increase of the queuing time of the user access request, then, we define cache file placement factors to measure the suitability of cache nodes to replica placement.

The probability of access to files is proportional to popularity according to the definition of popularity of files. The higher the popularity, the greater the probability of access will be. Therefore, if we have a cache file *f_n_*, 1 ≤ *n* ≤ *N* whose copy number is *R_n_*, the popularity is $\xi_{n}$, and the size is *C_n_*. We place it into *R_n_* different cache nodes (a cache node can store only one copy of the same file), then the expected access load for each cache node is $\xi_{n}/R_{n}$. Thus, if the cache node *Cache_l_* currently stores *N_l_* cache files, and the number of replica of the cache file *f_m_*, 1 ≤ *m* ≤ *N_l_* is *R_m_*, then the current expected access load of the cache node *Cache_l_* can be expressed as:

(6) $$Load_{l}=\sum_{m=1}^{N_{l}}\xi_{m}/R_{m}$$

Obviously, the smaller the expected access load of the cache node, the greater the cache capacity, and the wider the real-time bandwidth, the better it will be after the cache file is placed. Therefore, we define the cache file placement factor as follows:

(7) $$\rho_{l}=CS_{l}^{re}\times B_{l}/Load_{l}$$

where the cache capacity of the cache node *Cache_l_* is *CS_l_*, the remaining cache capacity is $CS_{l}^{re}=CS_{l}-\sum_{i=1}^{N_{l}}C_{l}$, and the real-time bandwidth is *B_l_*. Of course, the larger the cache file placement factor, the better it is for placing cache files.

With the cache file placement factor, we can place cache files according to the size of the current placement factor of each cache node. The placement principle is according to geographical area meshing code, each cache file in the grid area is placed one by one from the first grid area. The access associated files are placed first, followed by the access non-associated files. In addition, large files are placed first irrespective of whether the access is associated or not, then small files are placed, and the replica of the same file are place in different cache nodes. The steps for placing cache files are listed as follows.

*Step 1*: Place access associated filesets
  (1) Calculate the size of each fileset $F_{1}^{\prime }$.
  (2) Calculate the file placement factor for each cache node.
  (3) Place the first replica of the filesets with the largest size in $F_{1}^{\prime }$ as a whole in the cache node where the placement factor is largest. Then, if the storage capacity of the node is not enough, the cache node with the second-largest placement factor will be selected.
  (4) Loop steps (2–3), place the remaining replica in different cache nodes in turn.
  (5) Loop steps (1–4), place all access associate filesets in $F_{1}^{\prime }$ as a whole into the cache node.
*Step 2*: Place access non-associated filesets.
  (1) Calculate the size of each file in *F*_2_.
  (2) Calculate the file placement factor for each cache node.
  (3) Place the first replica of the filesets with the largest size in *F*_2_ into the cache node whose placement factor is the largest. Then, if the storage capacity of the node is not enough, select the cache node with the second-largest placement factor.
  (4) Loop steps (2–3), place the remaining replica in different cache nodes in turn.
  (5) Loop steps (1–4), place all files in *F*_2_ into the cache node.

Through the above steps, we can place all cache files in $F_{cache}=F_{1}\cup F_{2}=F_{1}^{\prime }\cup F_{2}$ to different cache nodes in the form of access associate or non-associated.

## 5. Experimental and Performance Evaluation

In this section, we will first introduce the performance evaluation metrics for our replication placement strategy. Then, the experimental data and methods will be described. Finally, we will present and discuss the results of the experiments.

### 5.1. Evaluation Metrics

Three indexes will be used during the experiment to evaluate the performance of the RSSD algorithm: cache hit rate, mining time, and average response time. These are defined as follows:

- Cache hit rates: a percentage of the total number of requests hit in a user access request, which is used to measure the performance of cache file selection mechanisms.
- Mining time: the time consumed for mining the access associated files from the historical user access information, which is used to measure the computational efficiency of the algorithm.
- Average response time: the average response time of user access to a single file, which is used to measure the overall performance of the algorithm.

### 5.2. Experimental Data and Methods

The experimental data was obtained from the Wuhan smart city network application demonstration platform, which includes 14 types of sensors located in different regions. It has been collecting sensor data since 1 January 2010, and provides 20 types of predefined applications to the public. The distributed cache system is a centralized topological network comprising the dispatcher, proxy server, and cache server, and built on top of the Hadoop distributed file system (HDFS). The cache system and the HDFS are loosely coupled. The scheduling proxy server is considered as a central index server while undertaking the request scheduling. Its internal storage has a global index table used for recording information of all nodes where the file cached, and the cache node also holds the local index. The cache node and the scheduler proxy server are connected through the 1000 M Ethernet switch, and the cache nodes are in non-communication with each other.

We obtained the user access logs in the server for the period from 1 March 2017 to 1 May 2017. After processing, we generated 976,328 file access requests, the size of these accessed files was approximately 843.6 GB. In order to calculate the popularity of the files, we converted the user access time to observation time *T* = [*t_begin_*:*t_end_*] = [1:1440], in hours, and set the time interval Δ*t* = 24, then divided the observation time *T* into *N_t_* = 60 segments. Based on this foundation, we can use the RSSD algorithm proposed in this paper to place cache replica files in different cache nodes.

### 5.3. Experimental Results

#### 5.3.1. Cache Hit Rates

**Figure 3:**
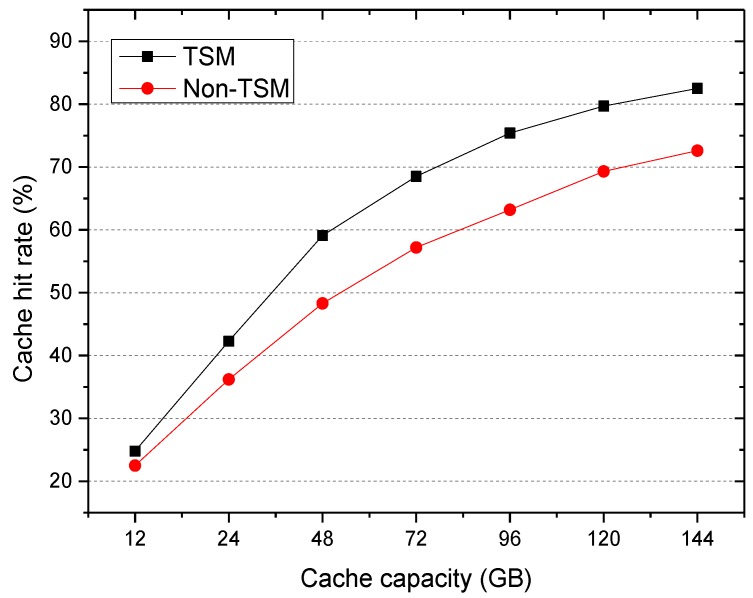
Cache hit ratio.

The experiment investigated the impact of two cache selection mechanisms on cache hit rate. The first did not consider the influence of the access time on the popularity, such as the number, frequency, and probability of file access in [6,7]. We called it the non-time-weighted selection mechanism (Non-TSM). The second considered the influence of the access time on the popularity, such as the nearest access maximum weight selection mechanism in [10], the time forgetting function selection mechanism in [11], and the RSSD algorithm proposed in this paper. We called it the time-weighted selection mechanism (TSM). The experimental results are shown in Figure 3.

As can be observed, as the cache capacity increased, the cache hit rate of the system gradually increased until it was stable. In addition, the TSM cache hit rate was higher than that of the Non-TSM, and this became more obvious as the cache capacity increased. This is because the Non-TSM mechanism directly calculates the number of accesses to the file, and the attenuation of file access over time is ignored.

#### 5.3.2. Mining Time

**Figure 4:**
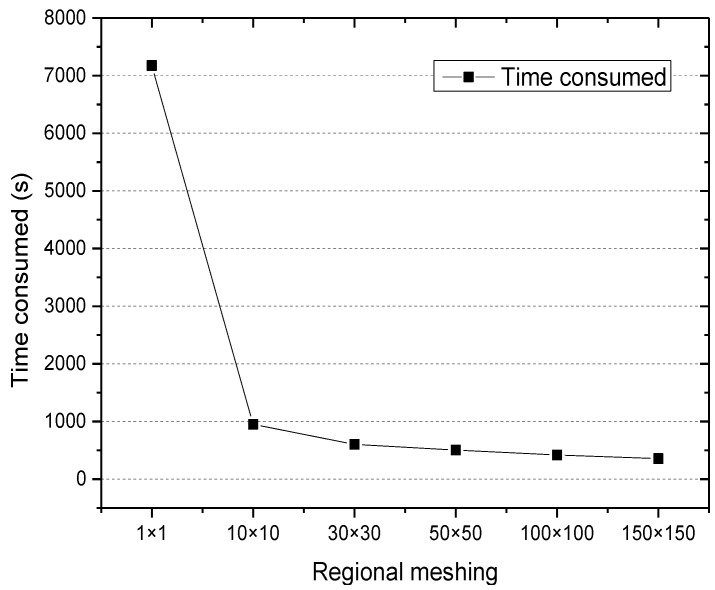
Time consumed.

The experiment investigated the time consumed for historical user access information under different regional meshing levels, that is, the influence of the regional meshing on the execution efficiency of the algorithm. The results are shown in Figure 4.

Figure 4 shows that the time consumed decreased when the regional meshing levels became larger. The time consumed in mining 676,328 file access information was 7164 s while the regional meshing level was 1 × 1. However, when the meshing level was 150 × 150, the time was 356 s. This is because regional meshing decomposition of the correlation calculation of files is changed from the entire geographic region to the regional cell, and the local and incremental calculation for file access associated is realized.

#### 5.3.3. Average Response Time

The total memory capacity of the distributed cache system in the following experiment was set to be 144 GB. A total of 50, 100, 200, 500, and 1000 accesses to different application services were randomly generated, aimed at testing the influence of regional meshing, cache file placement, and the number of user concurrent accessing to average response time.

(1) The influence of regional meshing on the average response time.

**Figure 5:**
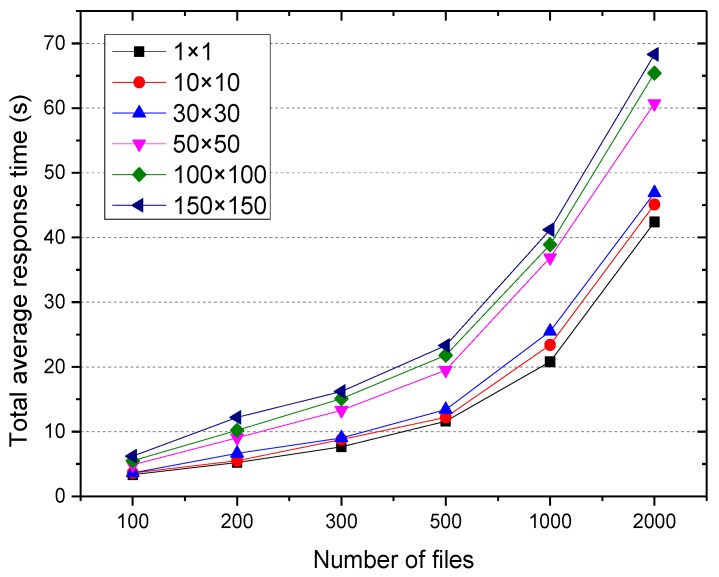
Total average response time.

The files belonging to the same time period, the adjacent geographic region, and high popularity were considered to be access associated. Therefore, regional meshing will impact the access associate between files, and also impact the average response time. Figure 5 shows the total average response time of users when accessing 500–3000 files by using application services in different regional meshing levels.

As can be observed, for the same regional meshing level, the total average response time increased as the number of file increased. However, for the same number of files, as the meshing level increased, the total average response time also increased, and the minimum was achieved when the meshing level was 1 × 1. This is because the increased density of the mesh, the lower probability of the user access files belonging to the adjacent geographic region and the same time period, and the less access associated files found cannot reduce the request cross node scheduling times.

(2) The influence of cache file placement on the average response time.

By combining the experimental results of executive efficiency and the average response time, it can be seen that the time consumed and the average response time were smaller when the regional meshing level was 30 × 30. Therefore, in the following experiment, we set the regional meshing level to be 30 × 30.

**Figure 6:**
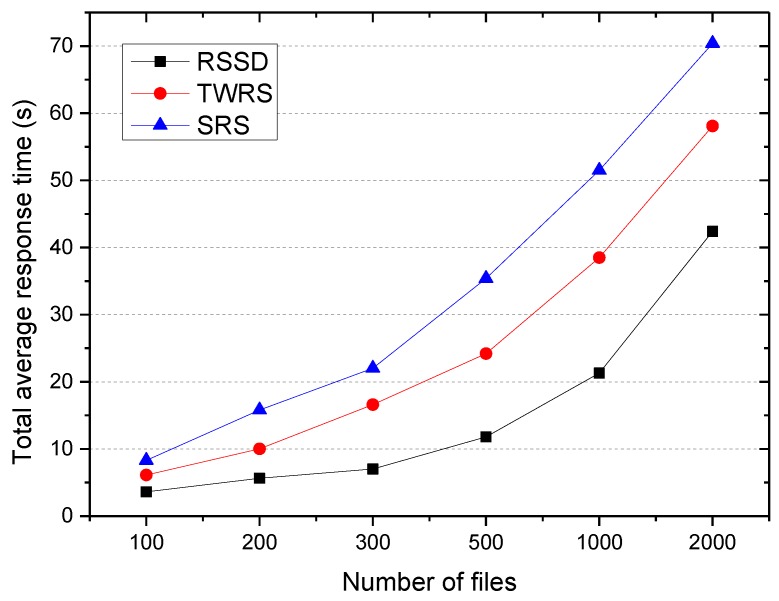
Total average response time.

In terms of the experimental comparison of the RSSD algorithm, the time-weighted replication strategy TWRS in [10,11] considered the decay time but did not consider access correlation, and the simple replication strategy SRS in [8,9,14] did not consider access correlation and decay time, but considered the access frequency, node load, real-time bandwidth, etc. The experiment investigated the total average response time of the system when the user accesses the 500–3000 files, and the result is shown in Figure 6.

It can be seen that the RSSD algorithm had the shortest response time, followed by TWRS, while SRS had the longest time. This is because the average response time consists of two parts: the queuing time and the cross node scheduling time. The TWRS algorithm only considers the time decay without considering access correlation, which can guarantee the cache hit ratio and load balance of the system, reduce the queue time of the request, but cannot reduce the cross-node scheduling time of the request.

The SRS algorithm places the cache files through the current load of the cache node, which can reduce the request queuing time with load balancing, but can neither guarantee cache hit rate nor reduce the number of cross node scheduling of requests such that the total average response time is the longest.

(3) The influence of concurrent accessing on the average response time.

**Figure 7:**
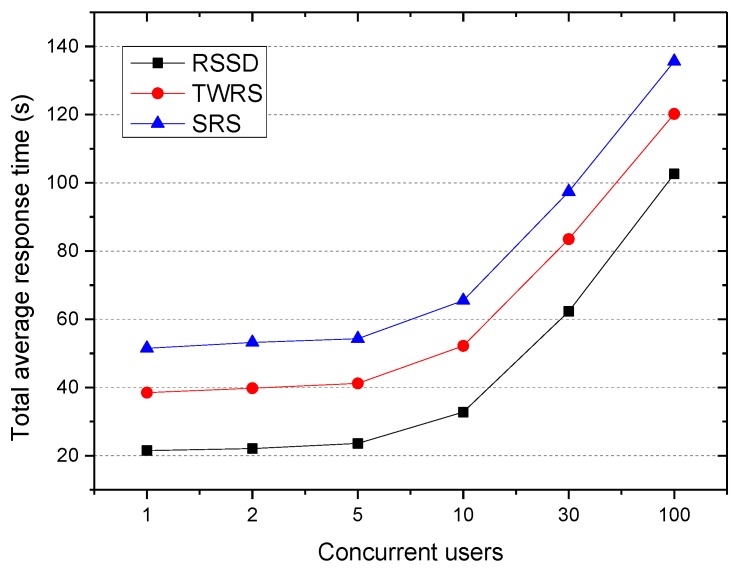
Total average response time.

The experiment compared the replica algorithm of RSSD, TWRS, and SRS, and investigated the total average response time of a user accessing 1000 files concurrently through predefined applications in a smart city. The result is shown in Figure 7.

It can be seen that as the number of concurrent access users increased, the average response time also increased; however, the total average response time of RSSD was still the smallest. This is because the RSSD not only creates replica, but also places the associated files as a whole in the same cache node and realizes bidirectional optimization of the request queuing and cross node scheduling times. In addition, it can also be seen from the graph that when the number of concurrent access users was 1–5, the total average response time of RSSD, TWRS, and SRS algorithms were almost unchanged and appeared to be stable, then increased rapidly. This is because the distributed cache node was set to be 6, and the number of replica of the cache file did not exceed the number of nodes; therefore, when the number of concurrency access of the same file was greater than 6, there was requests queuing, which increased the total average response time.

## 6. Conclusions and Future Work

The RSSD algorithm makes full use of the spatiotemporal locality and correlation of user access in a smart city, improves the cache hit ratio, and achieves bidirectional optimization of the queuing time and the number of cross node scheduling. RSSD is designed for cluster environment with distributed multi nodes. In the future, we will apply this method to the Hadoop distributed file system for the purpose of improving access performance.

## References

[1] Li D.R., Cao J.J., Yuan Y. (2015). Big Data in Smart Cities. Sci. China Inf. Sci..

[2] Li D.R., Yao Y., Shao Z.F., Wang L. (2014). From digital Earth to Smart Earth. Chin. Sci. Bull..

[3] Qin X.L., Zhang W.B., Wei J., Wang W., Zhong H., Huang T. (2013). Progress and Challenges of Distributed Caching Techniques in Cloud Computing. J. Softw..

[4] Zhang J., Wu G., Hu X., Wu X. A Distributed Cache for Hadoop Distributed File System in Real-Time Cloud Services. Grid Computing (GRID). Proceedings of the 2012 ACM/IEEE 13th International Conference on Grid Computing (GRID).

[5] Xiong L., Xu Z., Wang H., Jia S., Zhu L. (2016). Prefetching scheme for massive spatiotemporal data in a smart city. Int. J. Distrib. Sens. Netw..

[6] Tang M., Lee B.S., Yeo C.K., Tang X. (2005). Dynamic Replication Algorithms for the Multi-tier Data Grid. Futur. Gener. Comput. Syst..

[7] Tang M., Lee B.S., Tang X., Yeo C.K. (2006). The Impact of Data Replication on Job Scheduling Performance in the Data Grid. Futur. Gener. Comput. Syst..

[8] Sun X., Li Q.Z., Zhao P., Wang K.X., Pan F. (2014). An Optimized Replica Distribution Method for Peer-to-Peer Network. Chin. J. Comput..

[9] Li R., Feng W., Wu H., Huang Q. (2014). A Replication Strategy For a Distributed High-speed Caching System Based on Spatiotemporal Access Patterns of Geospatial Data. Comput. Environ. Urban Syst..

[10] Chang R.S., Chang H.P. (2008). A dynamic data replication strategy using access-weights in data grids. J. Supercomput..

[11] Sun D.W., Chang G.R., Gao S., Jin L., Wang X. (2012). Modeling a Dynamic Data Replication Strategy to Increase System Availability in Cloud Computing Environments. J. Comput. Sci. Technol..

[12] Xu X., Yang C., Shao J. (2017). Data Replica Placement Mechanism for Open Heterogeneous Storage Systems. Procedia Comput. Sci..

[13] Pan S., Xiong L., Xu Z., Meng Q. (2017). A dynamic replication management strategy in distributed GIS. Comput. Geosci..

[14] Wei Q., Veeravalli B., Gong B., Zeng L., Feng D. CDRM: A Cost-Effective Replication Management Scheme for Cloud Storage Cluster. Proceedings of the 2010 IEEE International Conference on Cluster Computing (CLUSTER).

[15] Li W.Z., Chen D.X., Lu L.S. (2010). Graph-Based Optimal Cache Deployment Algorithm for Distributed Caching Systems. J. Softw..

[16] Tu M., Yen I.L. (2014). Distributed replica placement algorithms for correlated data. J. Supercomput..

[17] Zaman S., Grosu D. (2011). A Distributed Algorithm for the Replica Placement Problem. IEEE Trans. Parallel Distrib. Syst..

[18] Nagarajan V., Mohamed M.A.M. (2017). A prediction-based replication strategy for data-intensive applications. Comput. Electr. Eng..

[19] Lin J.W., Chen C.H., Chang J.M. (2013). QoS-Aware Data Replication for Data-Intensive Applications in Cloud Computing Systems. IEEE Trans. Cloud Comput..

[20] Shorfuzzaman M., Graham P., Eskicioglu R. Distributed Placement of Replica in Hierarchical Data Grids with User and System QoS Constraints. Proceedings of the 2011 International Conference on P2P, Parallel, Grid, Cloud and Internet Computing (3PGCIC).

[21] Cheng C.W., Wu J.J., Liu P. (2009). QoS-aware, access-efficient, and storage-efficient replica placement in grid environments. J. Supercomput..

[22] You K., Tang B., Qian Z., Lu S., Chen D. (2013). QoS-aware placement of stream processing service. J. Supercomput..

[23] Tos U., Mokadem R., Hameurlain A., Ayav T., Bora S. (2015). Dynamic Replication Strategies in Data Grid Systems: A Survey. J. Supercomput..

[24] Suciu G., Butca C., Dobre C., Popescu C. Smart City Mobility Simulation and Monitoring Platform. Proceedings of the 2017 21st International Conference on Control Systems and Computer Science.

[25] Srikant R., Agrawal R. Mining Sequential Patterns: Generalizations and Performance Improvement. Proceedings of the 5th International Conference on Extending Database Technology: Advances in Database Technology.

[26] Han J.W., Pei J., Yin Y. Mining frequent patterns without candidate generation. Proceedings of the 2000 ACM SIGMOD International Conference on Management of Data (SIGMOD'00).

